# Targeting the TR4 nuclear receptor-mediated lncTASR/AXL signaling with tretinoin increases the sunitinib sensitivity to better suppress the RCC progression

**DOI:** 10.1038/s41388-019-0962-8

**Published:** 2019-09-09

**Authors:** Hangchuan Shi, Yin Sun, Miao He, Xiong Yang, Michiaki Hamada, Tsukasa Fukunaga, Xiaoping Zhang, Chawnshang Chang

**Affiliations:** 1 Department of Urology, Union Hospital, Tongji Medical College, Huazhong University of Science and Technology, Wuhan, 430022 China; 2 Radiation Oncology, The Wilmot Cancer Institute, University of Rochester Medical Center, Rochester, NY 14642 USA; 3 Faculty of Science and Engineering, Waseda University, Tokyo, 169-8555 Japan; 4 Sex Hormone Research Center and Department of Urology, China Medical University/Hospital, Taichung, 404 Taiwan; 5 Computational Bio Big-Data Open Innovation Laboratory (CBBD-OIL), National Institute of Advanced Industrial Science and Technology (AIST), Tokyo, 169-8555 Japan

**Keywords:** Cancer, Cell signalling, Cancer, Cell signalling

## Abstract

Renal cell carcinoma (RCC) is one of the most lethal urological tumors. Using sunitinib to improve the survival has become the first-line therapy for metastatic RCC patients. However, the occurrence of sunitinib resistance in the clinical application has curtailed its efficacy. Here we found TR4 nuclear receptor might alter the sunitinib resistance to RCC via altering the TR4/lncTASR/AXL signaling. Mechanism dissection revealed that TR4 could modulate lncTASR (ENST00000600671.1) expression via transcriptional regulation, which might then increase AXL protein expression via enhancing the stability of AXL mRNA to increase the sunitinib resistance in RCC. Human clinical surveys also linked the expression of TR4, lncTASR, and AXL to the RCC survival, and results from multiple RCC cell lines revealed that targeting this newly identified TR4-mediated signaling with small molecules, including tretinoin, metformin, or TR4-shRNAs, all led to increase the sunitinib sensitivity to better suppress the RCC progression, and our preclinical study using the in vivo mouse model further proved tretinoin had a better synergistic effect to increase sunitinib sensitivity to suppress RCC progression. Future successful clinical trials may help in the development of a novel therapy to better suppress the RCC progression.

## Introduction

Renal cell carcinoma (RCC) is one of the most lethal urological tumors with a worldwide mortality of about 90,000 annually based on the WHO statistics [1]. Although there is a higher incidence of detection of small renal masses through improved diagnosis, close to one-third of patients will eventually develop metastatic lesions in the course of the disease [2]. Drugs targeting vascular endothelial growth factor (VEGF) or mammalian target of rapamycin pathway have shown robust clinical effects and changed paradigm of treatment of metastatic RCC [3]. In particular, sunitinib, being able to bring significant prolongation of progression-free survival in patients, has become the first-line medicine in the systematic therapy for metastatic RCC. However, inherent or acquired resistance to these approaches have been found in most patients [4]. Thus far there is no clear understanding of the molecular mechanism responsible for this therapy resistance [5, 6].

Hypoxia is a frequent occurrence in a solid tumor mass, which has functional consequences for tumor progression [7], and RCC is a hypoxia-driven malignancy [8]. As reported, about 60–80% of RCC patients have loss-of-function coding mutations in the Von Hippel-Lindau (VHL) gene and histological necrosis, suggesting that hypoxia plays critical roles in the progression of RCC [9]. The main target of sunitinib is the vessels that nourish the tumor cells, thus sunitinib application will likely generate hypoxic conditions for the tumor cells. At the same time, sunitinib will also inhibit receptor tyrosine kinases in the RCC cells, and overall resistance to sunitinib likely will derive from the combined effects on vessels and on tumor cells. For practical reasons, we will examine the molecular mechanisms of sunitinib resistance in RCC cells with a focus on the role of Testicular orphan receptor 4 (TR4) under hypoxic conditions compared with normoxic conditions.

TR4 (nuclear receptor subfamily 2, group C, member 2) is a member of the nuclear receptor superfamily and encodes a 67-kDa protein [10]. Like other nuclear receptors, TR4 is a ligand-activated transcription factor. The activated receptor/ligand complex is translocated to the nucleus where it binds to hormone response elements of target genes [11]. Early studies showed that the protein encoded by TR4 functions in many biological processes in prostate cancer [12]. But its role in RCC is still not well understood.

AXL belongs to the Tyro3-Axl-Mer (TAM) family of receptor tyrosine kinases with growth arrest-specific 6 (GAS6) protein as the common ligand. GAS6/AXL stimulates multiple pro-tumorigenic signalings associated with tumor progression such as cellular adhesion, invasion, migration, proliferation, and anti-apoptosis. Recently, AXL signaling was found essential for VEGF-mediated activation of PI3K/AKT and migration of endothelial cells, suggesting that overexpression of AXL may serve as a mechanism of resistance to anti-angiogenic therapies [13].

Long noncoding RNAs (lncRNAs) over 200 bp in length have been found to be important for numerous molecular processes [14]. An increasing number of lncRNAs have been reported to be involved in RCC progression [15]. However, the proven roles of lncRNAs involved in RCC resistance to sunitinib are limited [16].

Tretinoin, a derivative of Vitamin A, can function via targeting the retinoic acid receptor alpha (RARα)-mediated signals to alter the cell apoptosis [17], and has been used as a medication to treat acute promyelocytic leukemia (APL) for decades [18]. However, its linkage to altering the TR4-mediated signals remains unclear.

Here we demonstrate that hypoxia increases TR4, which plays a critical role in regulating RCC resistance to sunitinib through lncTASR (ENST00000600671.1) that in turn regulates AXL. Thus, we delineated a novel pathway for TR4’s effects on the sunitinib resistance as well as provide a mechanistic explanation, and more importantly, we provided potential therapeutic approaches to overcome this resistance.

## Results

### Hypoxia treatment can increase RCC resistance to sunitinib via upregulating TR4 expression

**Fig. 1:**
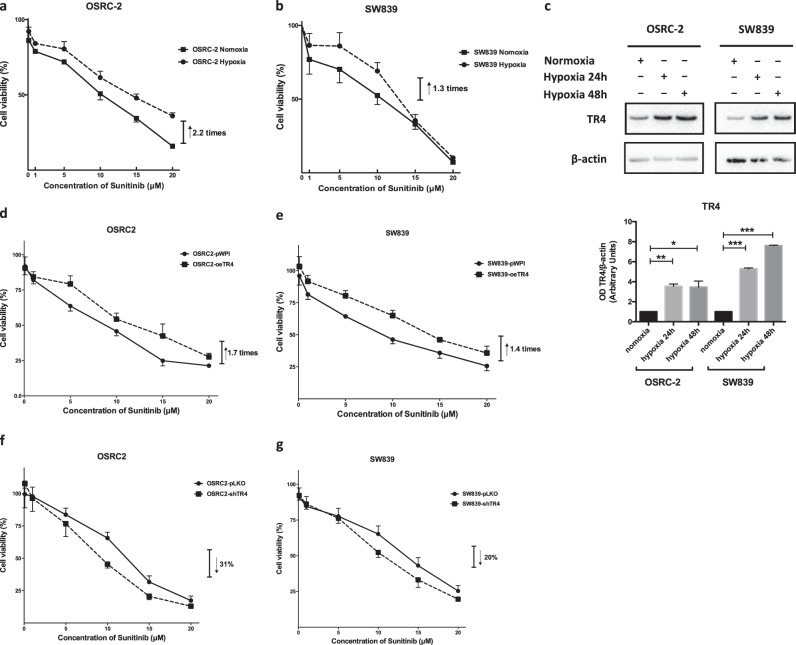
Hypoxia treatment can influence renal cell carcinoma sensitivity to sunitinib though regulating TR4. After hypoxia treatment, RCC cell lines OSRC-2 (**a**) and SW839 (**b**) show increased sunitinib resistance compared with that under normoxia conditions. The cells were cultured with different concentration gradients of sunitinib and harvested after 24 h treatment. (**c**) Western blot (upper) with quantification (lower) shows that TR4 expression increases in RCC cell lines after hypoxia treatment compared with that under normoxia conditions. The sunitinib resistance can be increased by introducing overexpressed TR4 in OSRC-2 (**d**) and SW839 (**e**) cells. The sensitivity to sunitinib can be increased by knocking down TR4 in OSRC-2 (**f**) and SW839 (**g**) cells. Data is presented as mean ± s.e.m., *p < 0.05, **p < 0.01, ***p < 0.001

As early studies indicated that hypoxia might impact the progression of several solid tumors including RCC [7, 19, 20], we were interested to see if hypoxia may also influence the RCC proliferation under sunitinib treatment. As shown in Fig. 1a, b, we found hypoxia treatment could increase RCC sunitinib resistance in RCC OSRC-2 and SW839 cells at 5 and 10 μM. We also calculated the IC_50_ of sunitinib in OSRC-2 cells at 6.5 μM.

As recent studies indicated that TR4 could also impact RCC progression [21, 22], we were interested to see if hypoxia may function via altering TR4 to influence the sunitinib sensitivity. The results revealed that hypoxia could significantly increase TR4 expression in both RCC OSRC-2 and SW839 cells (Fig. 1c). Importantly, interruption approaches with MTT assay also revealed that increasing TR4 expression could promote RCC sunitinib resistance (Fig. 1d, e), while suppressing TR4 via adding TR4-shRNA resulted in a decrease in the sunitinib resistance in OSRC-2 and SW839 cells (Fig. 1f, g). Furthermore, we evaluated TR4’s role in the RCC sunitinib resistance after hypoxia treatment. The results revealed that after the treatment of hypoxia, only knocking down TR4 led to increase sunitinib sensitivity of OSRC-2 and SW839 cell lines, but additional TR4 likely failed to exert an impact beyond the hypoxia-induced TR4 (Supplementary Fig. 1).

Taken together, results from Fig. 1a–g suggest that hypoxia may increase the RCC sunitinib resistance in RCC cells through regulating TR4 expression.

### Mechanism dissection of how TR4 can influence RCC sunitinib resistance: via altering AXL expression

**Fig. 2:**
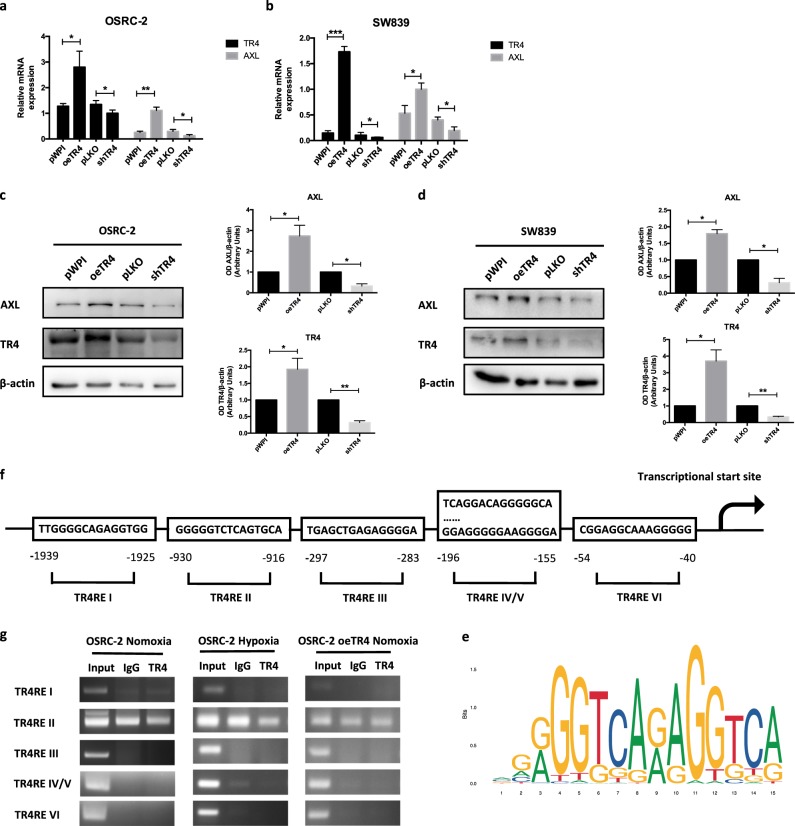
TR4 can influence renal cell carcinoma sensitivity to sunitinib via altering AXL expression. Under TR4 overexpressed and knocked down conditions, the expression of AXL changes along with TR4 at mRNA level in OSRC-2 (**a**) and SW839 (**b**) cells. Similar results are shown in protein level by Western blot (left) with quantifications (right) in OSRC-2 (**c**) and SW839 (**d**) cells. **e** TR4 binding profile map predicted by JASPAR. **f** Speculated potential promoter structure of AXL binding to the TR4 response element (TR4RE). **g** ChIP assay shows the binding capacity of TR4REs to the speculated promoter structure of AXL under the condition of normoxia, hypoxia and TR4 overexpression in OSRC-2 cells. In **a** and **b**, data is presented as mean ± s.e.m., *p < 0.05, **p < 0.01

To dissect the molecular mechanism of how hypoxia-induced TR4 can alter the RCC sunitinib resistance, we examined several genes that have been reported to play important roles on altering sunitinib sensitivity [4, 6, 16]. We then focused on the AXL, a key player in regulating the sunitinib resistance [13, 16], and results revealed that increasing the TR4 expression with TR4-cDNA or suppressing TR4 with TR4-shRNA altered the AXL mRNA expression in both RCC OSRC-2 and SW839 cells (Fig. 2a, b). Western blot analysis also confirmed alteration of TR4 protein expression changed the expression of AXL in both OSRC-2 and SW839 cells (Fig. 2c, d).

To further dissect the molecular mechanism of how TR4 can alter the AXL expression, we then applied the bioinformatics analysis to characterize the potential promoter structure of AXL (Fig. 2e, f) and tested TR4’s binding to the potential TR4 response elements (TR4REs). However, results from the Chromatin immunoprecipitation (ChIP) in vivo binding assay with anti-TR4 antibody revealed that TR4 failed to bind to those potential TR4REs located on the AXL 5′ promoter region in OSRC-2 cells, or OSRC-2 cells with overexpressed TR4, or OSRC-2 cells after hypoxia treatment (Fig. 2g).

Taken together, the results from Fig. 2a–g suggest that while TR4 may influence RCC sunitinib resistance via altering AXL expression, TR4 may not do so via direct transcriptional regulation.

### TR4 may modulate the AXL expression via altering the lncTASR expression

**Fig. 3:**
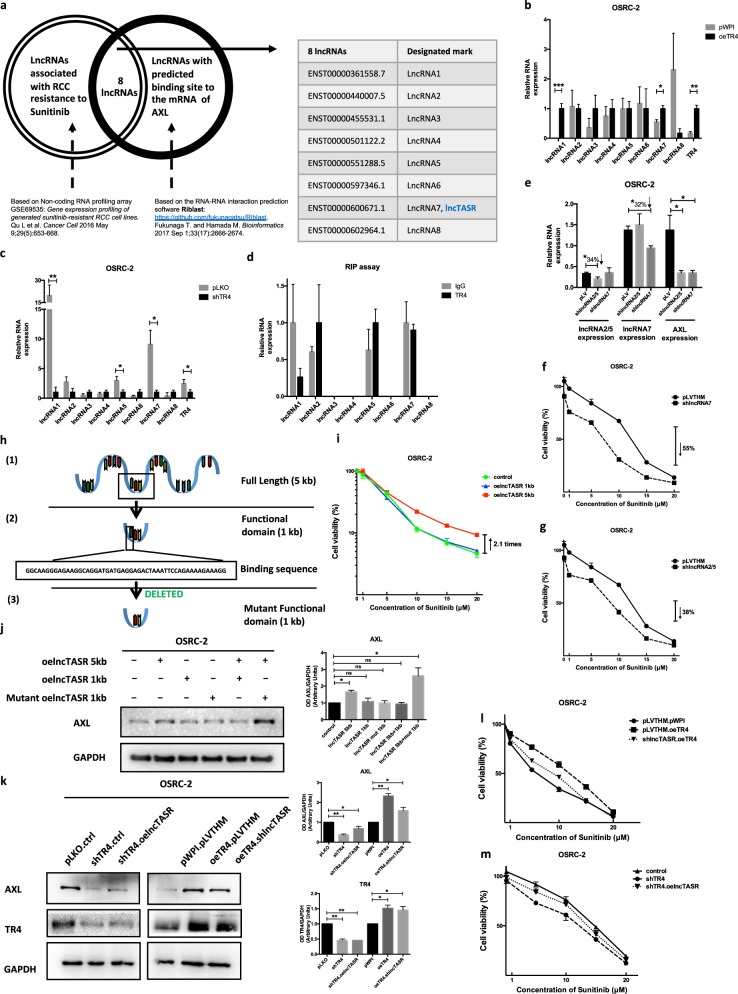
TR4 may alter AXL expression via altering lncTASR. **a** Bioinformatics analysis of potential lncRNAs that are both associated with RCC sunitinib resistance and have predicted binding sites to the mRNA of AXL. After TR4 was overexpressed (**b**) and knocked down in OSRC-2 cells (**c**), the expression of candidate lncRNA AC010300.1-201 (ENST00000600671.1, labeled as lncRNA7) changes along with TR4 at RNA level. **d** RIP assay identifies candidate lncRNAs EMX2OS-204 (ENST00000440007.5, labeled as lncRNA2) and EMX2OS-206 (ENST00000551288.5, labeled as lncRNA5), which can interact with TR4. These two lncRNAs are different splice variants of the transcript of EMX2OS and highly overlapped, so they are combined and labeled as lncRNA2/5. **e** AXL expression decreases at the mRNA level after knocking down lncRNA7 and lncRNA2/5. The sensitivity to Sunitnib can be increased by knocking down lncRNA7 (**f**) and lncRNA2/5 (**g**) in OSRC-2 cells. We named lncRNA7 lncTASR (lncRNA of TR4/AXL signaling related to sunitinib resistance) and **h** shows the predicted functional (1 kb) domain (2) of lncTASR, its full length (1) (5 kb) form and the constructed mutant (3) form of functional domain. **i** The sunitnib resistance can be increased only by introducing overexpressed full length lncTASR in OSRC-2 cells. **j** Western blot (left) with quantification (right) shows the expression of AXL when introducing different combinations of different lncTASR constructs. **k** The change of AXL expression caused by TR4 can be reversed by altering lncTASR. **l**, **m** The alteration of the RCC sensitivity to sunitinib influenced by TR4 can be reversed by lncTASR. In all but a and h, data is presented as mean ± s.e.m., *p *<* 0.05, **p *<* 0.01

We then examined if TR4 may alter the AXL expression via lncRNAs that could impact the progression of many tumors [16, 23]. We applied a bioinformatic RNA profiling array analysis to uncover the potential RNA–RNA interactions and identified eight lncRNAs candidates that might be able to interact with AXL and concurrently are highly related to sunitinib resistance in RCC (Fig. 3a).

Among these eight potential lncRNAs, we found the expression of lncRNA AC010300.1-201 (ENST00000600671.1, labeled as lncRNA7) and lncRNA MUC2-201 (ENST00000361558.7, labeled as lncRNA1) changed significantly after alterations of TR4 (Fig. 3b, c). As it is technically challenging to study lncRNA1, which is 12,667 kb in length, we decided to first focus on lncRNA7 for further characterization. In addition, results from the RIP assay also revealed that lncRNA EMX2OS-204 (ENST00000440007.5, labeled as lncRNA2) and lncRNA EMX2OS-206 (ENST00000551288.5, labeled as lncRNA5) could interact with TR4 (Fig. 3d). These two lncRNAs are different splice variants of the transcript of EMX2OS and highly overlapped, so they were combined and labeled as lncRNA2/5.

We then knocked down lncRNA7 and lncRNA2/5 in OSRC-2 cells and results revealed that suppressing these lncRNAs decreased the AXL mRNA expression (Fig. 3e), with increased RCC sensitivity to sunitinib by 55% at 10 μM (lncRNA7) and 38% at 10 μM (lncRNA2/5) (Fig. 3f, g). We decided to choose lncRNA7 (renamed as lncTASR [lncRNA of TR4/AXL signaling related to Sunitinib Resistance]) for further study, since its effect on changing the sunitinib resistance is more significant than that of lncRNA2/5.

The full length of lncTASR is 5 kb with a predicted binding motif of AXL located in the 5′ end of the lncRNA (Fig. 3h). It appears that only the full length lncTASR, not a one kilobase region containing the binding motif, is capable of regulating sunitinib sensitivity, suggesting that the binding alone is not sufficient to regulate AXL (Fig. 3i). We further demonstrated this finding at the protein level showing that the full length lncTASR, and not the 1 kb region (functional domain) of lncTASR can influence the expression of AXL (Fig. 3j).

To further characterize the functional structure of lncTASR, we designed a mutant functional domain of lncTASR (Fig. 3h), and then tested the expression of AXL by introducing different combinations of our three lncTASR constructs (full length, functional domain, and mutant functional domain). Interestingly, we found that if we introduced both functional domain and full length lncTASR, then the functional domain could compete with the full length lncTASR and interfere with the expression of AXL (Fig. 3j). These results suggest that 1 kb region containing the binding motif of lncTASR is necessary but not sufficient for regulating AXL expression by lncTASR.

Furthermore, we demonstrated that TR4 might modulate the AXL expression via altering the lncTASR. Figure 3k showed that lncTASR could reverse the TR4 impact on the AXL expression. Importantly, interruption *via* adding lncTASR or knocking down lncTASR could also reverse TR4's impact on the regulation of RCC sensitivity to sunitinib (Fig. 3l, m). It appears that the lncTASR's regulation of sunitinib sensitivity and AXL expression are not completely linear in that its impact on sunitinib sensitivity is more significant than on AXL expression (Fig. 3i and k). It is possible that lncTASR may regulate targets other than AXL, but the exact molecular details remain to be examined.

Together, results from Fig. 3a–m suggest that TR4 may function via lncTASR to alter AXL expression.

### Mechanism dissection of how TR4 can modulate lncTASR expression: via transcriptional regulation

**Fig. 4:**
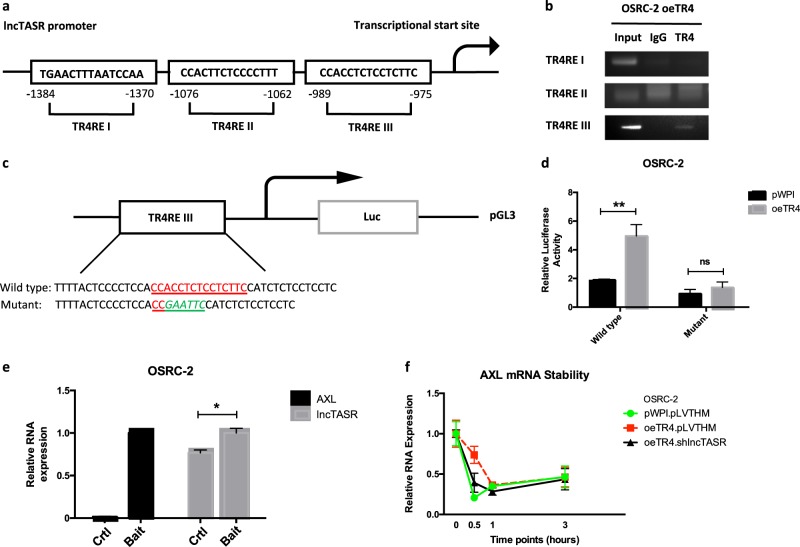
Mechanisms of the TR4/lncTASR/AXL axis. **a** Speculated potential promoter structure of lncTASR binding to the TR4 response element (TR4RE). **b** ChIP assay shows the binding capacity of TR4RE to the speculated promoter structure of lncTASR. **c** Diagram of the cloned 1.5 kb lncTASR promoter into pGL3 basic luciferase report vector (pGL3-Wt). Site-directed mutagenesis of TR4RE by mutating part of the TR4RE to EcoRI cutting site (-GAATTC-). **d** OSCR-2 cells were co-transfected with wild type or mutant lncTASR promoter PGL3-Luciferase constructs and with/without oeTR4 and the luciferase assay was applied to detect the luciferase activity. **e** The RNA pull-down assay revealed that lncTASR can physically bind to AXL mRNA. A bait that can pull-down the mRNA of AXL was designed and the quantity of lncTASR was detected in the pull-down complex. **f** The AXL mRNA stability after altering TR4 and lncTASR. For **d**–**f**, data is presented as Mean ± s.e.m., *p < 0.05, **p < 0.01, ns = not significant compared with the controls

To further dissect the mechanism of how TR4 can modulate lncTASR expression, we examined whether TR4 could regulate lncTASR at the transcriptional level and identified three potential TR4REs on the 5′ promoter region of lncTASR (Fig. 4a). Results from ChIP assay with anti-TR4 antibody revealed that TR4 could bind to the TR4RE III on the 5′ promoter region of lncTASR in OSRC-2 cells (Fig. 4b).

We also constructed the lncTASR promoter luciferase reporter by inserting a 1.5 kb 5′ promoter region of lncTASR containing TR4RE III into the pGL3 luciferase backbone as well as a construct with the mutated TR4RE III (Fig. 4c). As expected, the luciferase assay results revealed that adding TR4 significantly increased luciferase activity in OSRC-2 cells transfected with wild type lncTASR promoter, but not in the cells with the mutant lncTASR promoter (Fig. 4d).

Together, results from Fig. 4a–d suggest that TR4 can modulate the lncTASR expression via transcriptional regulation.

### Mechanism dissection of how TR4-lncTASR axis can alter AXL expression: via binding to its 5′UTR region

Next, to dissect the mechanism how TR4-lncTASR can modulate the AXL expression, we applied the RNA pull-down assay and results revealed that lncTASR could bind directly to the 5′ UTR of the AXL mRNA (Fig. 4e). Results from mRNA stability assay also revealed that altering TR4-lncTASR led to the change of AXL mRNA stability (Fig. 4f).

Together, results from Fig. 4a–f suggest that TR4 could modulate the lncTASR expression via transcriptional regulation, which might then alter the AXL protein expression via enhancing the stability of AXL mRNA to increase the RCC sunitinib resistance.

### Human clinical data support the TR4/lncTASR/AXL axis in the RCC progression

**Fig. 5:**
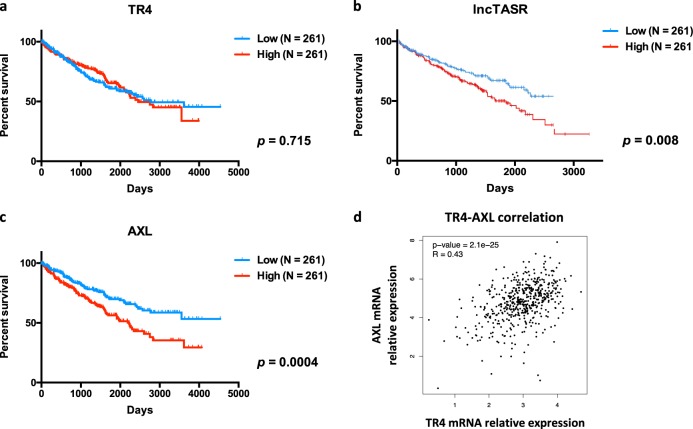
Clinical data support the TR4/lncTASR/AXL axis is critical for patients survival. **a** Clinical data from TCGA (*n* = 522) showing the relationship between the overall survival and expression of TR4. **b** Clinical data from OncoLnc (*n* = 522) showing the relationship between the overall survival and expression of lncTASR. **c** Clinical data from TCGA showing the relationship between the overall survival and expression of AXL. **d** Data analysis from TCGA database showing the correlation of TR4 and AXL. For **a**–**c**, data is presented as Mean ± s.e.m.

Human clinical data from TCGA and OncoLnc databases suggest that the RCC patients with higher expression of TR4, lncTASR, or AXL all have significantly shorter overall survival (Fig. 5a–c), and a positive correlation between expression of TR4 and AXL was also found from TCGA database [24] (*n* = 522) (Fig. 5d).

Together, results from human clinical sample surveys suggest that TR4/lncTASR/AXL signaling is critical for the survival of RCC patients.

### Targeting TR4/lncTASR/AXL signaling with small molecules including TR4-shRNA, tretinoin, or metformin all led to increase sunitinib sensitivity to better suppress the RCC progression

**Fig. 6:**
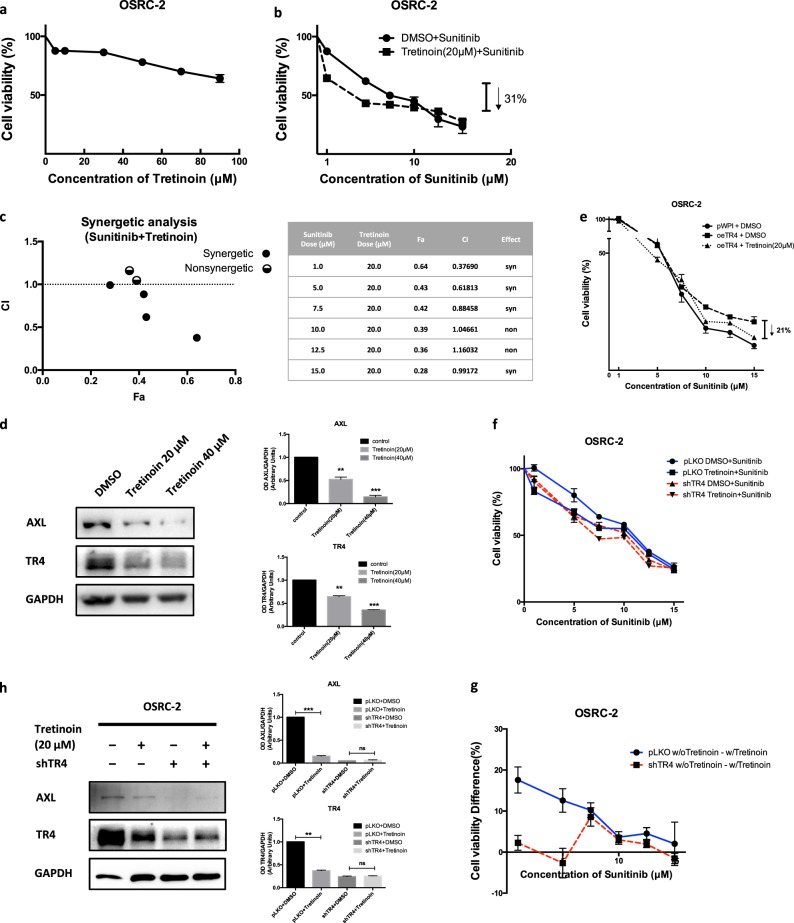
Targeting TR4/lncTASR/AXL signaling with tretinoin can increase sunitinib sensitivity to better suppress the RCC progression. **a** Tretinoin alone was not toxic to RCC cells. **b** Tretinoin can enhance the sensitivity of RCC to sunitinib when combined with low dose sunitinib. **c** Tretinoin actually has synergetic effects when combined with sunitinib in the treatment of RCC cells. **d** Western blotting (left) with quantification (right) shows that the expression of both TR4 and AXL decreased after treated with tretinoin. **e** Tretinoin can reverse the resistance of RCC cells to sunitinib caused by overexpressed TR4. **f** Viability of OSRC-2 cells in sunitinib with/without tretinoin and with/without TR4-shRNA. **g** DID (difference in difference) analysis shows that TR4-knockdown OSRC-2 cells have less difference change in sunitinib resistance with/without tretinoin compared with OSRC-2 cells without TR4-knockdown. **h** Tretinoin decreases both TR4 and AXL protein expressions in OSRC-2 cells with pLKO vehicle, but does not decrease TR4 and AXL expressions in TR4-knockdown OSRC-2 cells. Data is presented as Mean ± s.e.m., **p < 0.01, ***p < 0.001, ns not significant

To link the above findings to the future potential clinical application, we were interested to find if any small molecules can be used as possible therapeutic interventions to target this newly identified TR4/lncTASR/AXL signaling. Among several likely candidates, including TR4-siRNAs [25, 26], miRNAs [22], TR4 potential antagonists/agonists including tretinoin (Hu et al. unpublished data), and metformin [27] that can suppress the TR4 function, we found that adding TR4-shRNA (See Fig. 1f, g), metformin or tretinoin (up to 10 μM), could also result in increasing the sunitinib sensitivity to better suppress the RCC OSRC-2 cell growth (Fig. 6a, b, Supplementary Fig. 2a, b). Consistent with the biological effect of tretinoin and metformin, expression of tretinoin responsive genes *CRABP2* and *RARα* was increased by tretinoin while expression of *SREBP1c* increased in response to metformin (Supplementary Fig. 3).

Mechanism studies indicated that tretinoin (Fig. 6c) or metformin (Supplementary Fig. 2c) indeed have synergetic effects with sunitinib to better suppress the RCC viability. As expected, adding tretinoin (Fig. 6d) or metformin (Supplementary Fig. 2d) also led to suppress the expression of both TR4 and AXL in OSRC-2 cells. Interestingly, when we compared the impacts of these two small molecules on altering sunitinib sensitivity, we found that metformin could have synergetic effect with the high-dose of sunitinib (7.5–15 μM), yet tretinoin could have synergetic effect with low-dose of sunitinib (1.0–7.5 μM) in the OSRC-2 cells.

To further address whether the anti-sunitinib resistance function of tretinoin is through TR4 or other targets, we designed an assay to test the changes of sunitinib-sensitivity after TR4-knockdown in OSRC-2 cells with/without tretinoin, compared with OSRC-2 cells without TR4-knockdown. The results showed that Tretinoin has less synergetic effects with the low-dose of sunitinib (1.0–7.5 μM) on TR4-knockdown OSRC-2 cells (with less difference change compared with OSRC-2 without TR4-knockdown) (Fig. 6f, g). To confirm that tretinoin can regulate AXL expression and RCC sunitinib resistance mainly through TR4, we checked TR4 and AXL expressions in OSRC-2 cells with/without TR4-shRNA and with/without tretinoin. Our results in Fig. 6h showed that tretinoin decreases both TR4 and AXL protein expressions in OSRC-2 cells with pLKO control cells, but does not decrease TR4 and AXL expressions in TR4-knockdown OSRC-2 cells.

We also tested whether metformin plays a role in anti-Sunitnib resistance through TR4. We found that although metformin has less synergetic effects with the high-dose of sunitinib on TR4-knockdown OSRC-2 cells, metformin continues to decrease TR4 and AXL expressions in TR4-knockdown OSRC-2 cells (Supplementary Fig. 2f–h).

These results suggest that tretinoin can lead to increase sunitinib sensitivity through TR4 and AXL signaling, but we cannot conclude the anti-sunitnib resistance function of metformin is mainly through TR4. We therefore decided to choose tretinoin to test its in vivo roles to increase the sunitinib sensitivity.

### Preclinical study using in vivo mouse model to demonstrate targeting the TR4/lncTASR/AXL signaling using tretinoin can increase the RCC sensitivity to sunitinib

To confirm all above in vitro cell lines data in the in vivo nude mouse model, we performed the orthotopic left kidney capsule implantation using OSRC-2 cells labeled with firefly luciferase. After 14 days’ tumor development, we treated four groups of mice with vehicle control (DMSO), sunitinib (20 mg/kg), tretinoin (25 mg/kg) or both sunitinib and Tretinoin by intraperitoneal injection once every other day. The IVIS was used to monitor tumor growth every 7 days. After 28 days of drug treatment, we sacrificed the mice and examined the tumor formation and metastasis, and tumor weights from the abdominal and thoracic cavities.

**Fig. 7:**
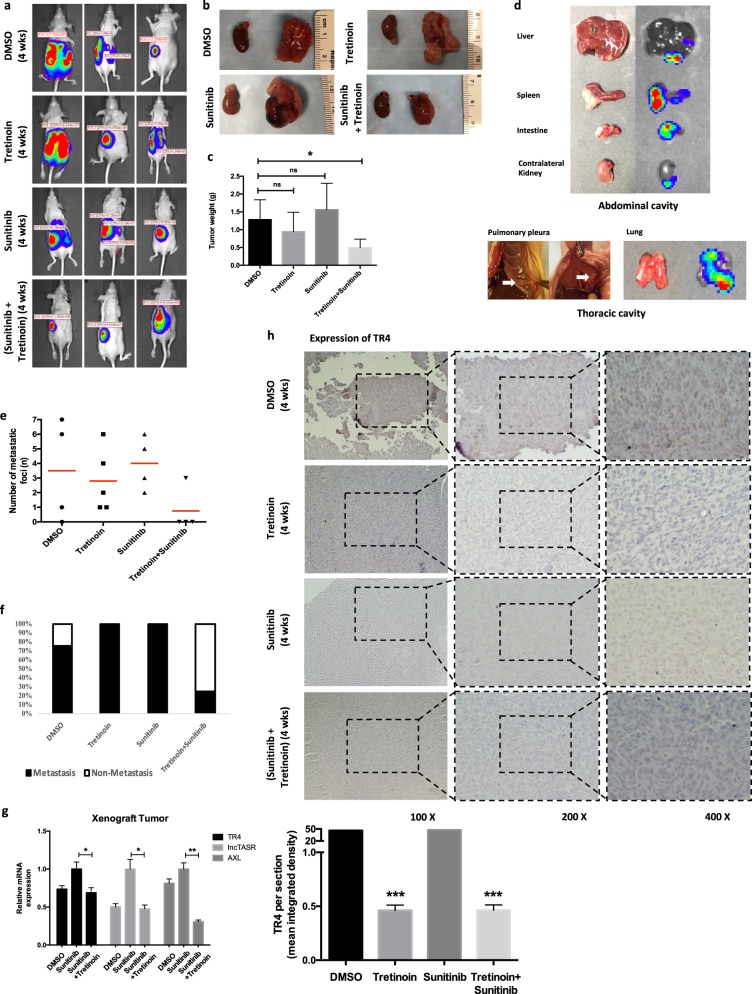
The TR4/lncTASR/AXL signaling in RCC resistance to sunitinib in xenograft mouse model. **a** IVIS imaging was used to determine the tumor luminescence value of representative mice over time. **b** Representative images of tumors from different groups. **c** After 28 days treatments (DMSO, sunitinib 20 mg/kg/q.d., or tretinoin 25 mg/kg/q.d., or both), the mice were sacrificed and the tumor weights were measured and calculated by the weight of the left kidney with tumor minus the weight of right normal kidney from the same mouse. **d** Representative photos and bioluminescent images of metastases in abdominal cavity and thoracic cavity. **e** Total metastatic foci of each mouse in four groups (**f**) and quantification for the mice metastasis ratio of each group. **g** Three tumor samples were randomly picked up from each group, the tissues were lysed and then the TR4, lncTASR and AXL level were detected by qRT-PCR. (**h**) The IHC staining (upper) with quantification (lower) to identify the level of TR4 in four groups. For c, and e–h, data is presented as mean ± s.e.m., *p *<* 0.05, **p *<* 0.01 compared with the controls

The IVIS mice images showed that the tretinoin + sunitinib group had a lower tumor luminescence value than the other three groups (Fig. 7a). After mice were sacrificed, the tumor image (Fig. 7b, the kidney with orthotopically planted tumor is on the right and the contralateral kidney from the same mouse is on the left) and tumor weight measurement (Fig. 7c) also confirmed this result. Then we counted the number of mice with metastases and metastatic foci in abdominal and thoracic cavities. The results revealed that a lower metastatic ratio and total metastatic foci were found in tretinoin + sunitinib group (Fig. 7d–f).

Notably, our results in Fig. 7a–f showed that the sunitinib group had no decreased tumor weight, metastatic ratio, and total metastatic foci compared with DMSO group. These are similar with the results from sunitinib-resistant mouse model established by Zhou et al. [13]. They injected 786-O RCC cells into nude mice to develop xenografts, continuously treated mice with sunitinib and observed that tumors regrew after 4 weeks sunitinib treatment. Our results also demonstrated that we successfully established an in vivo sunitinib-resistant model.

Importantly, results from qRT-PCR also confirmed our in vitro cell lines data showing TR4 can promote lncTASR expression in mice tumor samples to trigger the AXL axis and tretinoin can suppress this axis by targeting TR4 (Fig. 7g). In addition, results from IHC staining of TR4 confirmed that tretinoin could significantly decrease the expression of TR4 (Fig. 7h).

**Fig. 8:**
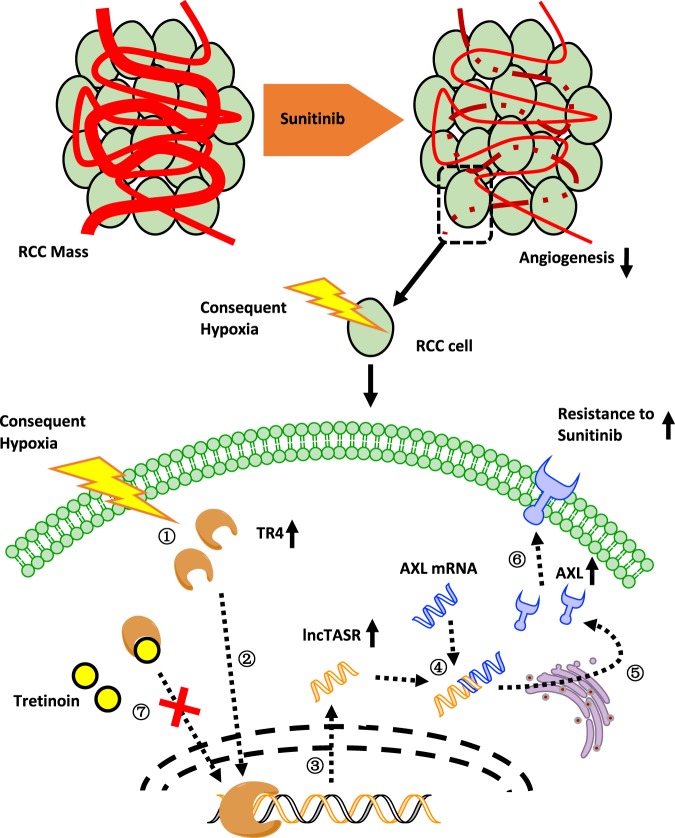
Mechanisms of TR4/lncTASR/AXL axis in RCC resistance to sunitinib and regulatory role of tretinoin. sunitinib treatment inhibits angiogenesis in RCC mass, making a consequent hypoxia condition around RCC cells. **①** Under hypoxia condition, TR4 expression increases. **②** Increased TR4 in nuclei plays a role as a transcriptional factor. **③** The expression of lncTASR increases under the regulation of TR4. **④** Increased lncTASR can stabilize AXL mRNA. **⑤** Increased stabilized AXL mRNAs are translated. **⑥** Increased AXL on the membrane stimulates alternative pathways to increase the resistance of RCC to sunitinib. **⑦** Tretinoin may bind to TR4 and degrade it to block the downstream of TR4, consequently reversing the resistance

Together, results from Fig. 7a–h mouse studies confirm our in vitro cell lines results showing that tretinoin can increase RCC sensitivity to sunitinib by targeting the TR4/lncTASR/AXL axis. The overall mechanisms of TR4/lncTASR/AXL axis in RCC sunitinib resistance and regulatory role of tretinoin are summarized in Fig. 8.

## Discussion

The development of anti-angiogenic drugs to treat RCC, such as sunitinib and Axitinib, are based on the finding of frequent inactivation of VHL in RCC [28]. Sunitinib achieved its inhibitory effect on tumor angiogenesis mainly by blocking VEGFR and PDGFR [29, 30], which results in a significant longer progression free survival (11 versus 5 months, *p* < 0.001) and overall survival (26.4 versus 21.8 months, *p* = 0.049) compared to interferon alpha (IFN-α) therapy [31]. However, RCC progression usually recurs after a median of 6–15 months of sunitinib therapy, suggesting the existence of intrinsic as well as acquired drug resistance [4].

Due to the difficulty in obtaining clinical samples from metastatic RCC patients, conventional in vitro studies on the resistance mechanisms were based on the establishment of sunitinib-resistant cell lines by chronic exposure of this drug. However, considering the pharmacologic mechanism of sunitinib, which mainly targets the VEGFR of the endothelial cells constructing the vessels around RCC solid tumors, the in vitro induced sunitinib resistance in RCC cells has weak clinical relevance. To approximate in vitro tissue culture cells to in vivo condition after sunitinib treatment, which results in a hypoxic tumor microenvironment, we attempted a comparison of the molecular changes of RCC cells under hypoxia versus normoxia conditions with a focus on TR4 protein. This is an extension of the recent study of another nuclear hormone receptor, androgen receptor, in its association with the acquired sunitinib resistance [32]. These studies indicate that nuclear hormone receptors can play a significant role in RCC resistance to sunitinib, raising the possibility of using small molecule hormones as adjuvant therapies for sunitinib.

Generally, the resistance mechanisms to tyrosine kinase inhibitors (TKIs) can be divided into several distinct groups including those affected by the tumor microenvironment, increased tumor invasiveness and metastasis, activation of alternative signaling pathways as well as insufficient target inhibition [6], and resistance mediated by the action of noncoding RNAs [16, 33, 34]. Among all those mechanisms, activation of alternative signaling pathways are the major focus and AXL, along with c-MET, were identified as important mechanisms of resistance of multiple tumors to TKIs [35, 36], such as non-small cell lung cancer to Gefitinib or Erlotinib [37], gastrointestinal stromal tumors to Imatinib [38], and HER2-positive breast cancer to Lapatinib [39]. Most recently, Qu et al. found that combined with BMS-777607, a small-molecule inhibitor of both c-MET and AXL, RCC cells could overcome the sunitinib resistance [16]. Moreover, Zhou et al. showed that Cabozantinib, a FDA proven TKI with multiple targets including c-MET and AXL [40], could rescue RCC sunitinib resistance both in vitro and in vivo [13]. In a Phase II study compared with sunitinib, cabozanitinib has been tested on metastatic RCC patients (NCT01835158) [41]. However, as a promising target to increase the sunitinib efficiency, there is limited understanding of how AXL is regulated, as well as its exact role in the resistance to TKI.

Our study identified a signaling axis, TR4/lncTASR/AXL, which is associated with the RCC sunitinib resistance. This is the first linkage of the regulation of AXL with TR4, a nuclear transcription factor, and may provide several potential targets to influence the RCC sunitinib resistance. In addition to the TR4-shRNAs or some selective miRNAs [22, 25, 26], we were more interested in using small molecules to target the TR4 function. As the structure of the TR4 ligand binding domain indicates that it might be activated by retinoic acids as that in RXRα [42], we find variants of retinol, including tretinoin, can regulate TR4 function. In addition, metformin, a currently used medicine to treat diabetes [43], was also found to be able to suppress the TR4 expression [27]. Indeed, our results revealed that both tretinoin and metformin could suppress the expression of TR4 and AXL, and increase the sunitinib sensitivity to better suppress the RCC cell growth (Fig. 6).

Early studies indicated that metformin at 5–50 mM could effectively suppress in vitro tumor growth in cells from lung cancer, prostate cancer, and kidney cancer [44–46]. Also, in vivo mice studies indicated that metformin at 250 mg/kg body weight (or 40 μM in plasma) could effectively suppress the colon cancer growth [47], yet none of the 23 currently ongoing human clinical trials has yielded any positive outcome in overall survival [48–50] with metformin.

In contrast, tretinoin, an analog of vitamin A, was first proven as a medication for acne [51], and has been used to treat APL since the 1990s [52]. Recent studies also reported its potential role on suppressing other cancers such as lung cancer [53] and thyroid cancer [54]. In this regard, our in vitro cell line results also revealed that tretinoin had synergetic effects with lower concentrations of sunitinib (Fig. 6a, b), a more achievable concentration than that for metformin to have synergistic effects with sunitinib (Fig. 6c, d).

Notably, we found that the nude mice treated with tretinoin became weaker than the ones without tretinoin. The weak mice had low body weight and thin muscles (Supplementary Fig. 4). The similar tretinoin in vivo adverse effect was also reported when combined with arsenic trioxide [55]. However, the weight loss did not seem to influence the tumor size. Mice from the tretinoin group grew similar size tumors than mice from the DMSO group, and mice from the tretinoin + sunitinib group grew the smallest tumor. It is possible that the modulatory effect of tretinoin on cytokines and lymphocytes may contribute to the overall weakness of the mice especially considering that nude mice are immunologically impaired [56, 57]. Future studies involving clinical use of tretinoin in RCC will benefit from close monitoring of the immune status of patients.

In conclusion, our study identified a TR4/lncTASR/AXL signaling pathway, which is associated with the RCC sunitinib resistance. Preclinical studies demonstrated that tretinoin can be a novel promising drug to overcome this RCC resistance by decreasing TR4 and AXL, establishing the foundation for further testing this combination in a clinical setting to enhance sunitinib efficacy in RCC in patients.

## Methods

### Cell culture

Human RCC cell lines OSRC-2 and SW839 were obtained from the American Type Culture collection (ATCC, Rockville, MD). Cells were cultured in DMEM media containing 1% penicillin and streptomycin, supplemented with 10% fetal bovine serum, in a 5% (v/v) CO_2_ humidified incubator at 37 °C.

### Hypoxia

Hypoxia (0.5% O_2_, 5% CO_2_, 94.5% N_2_) was achieved using an In Vivo2 hypoxic workstation (Ruskinn Technologies, Sanford, ME) or in a positive pressure chamber receiving gas from a custom-mixed tank (Airgas, Rochester, NY).

### MTT assay

Cell proliferation rates were measured using MTT. After being transfected with plasmids for 24 h, SW839 or OSRC-2 cells were seeded at 1000 per well in 96-well plate. The assay was performed after 24 h treatment of gradient concentrations of sunitinib with/without tretinoin. 0 μM means the same volume of DMSO as other concentration of sunitinib. MTT reagent was added to each well and then the plate was incubated for 2 h at 37 °C. Before the endpoint of incubation, we added DMSO to dissolve the ppt and the absorbance was measured at 570 nm. Each sample was assayed in triplicate and multiple times.

### RCC cell lines with differential stable expressions of TR4

The pWPI-TR4/pWPI-vec, or pLKO-shTR4#1/pLKO-shTR4#2/pLKO1-vec, the psPAX2 packaging plasmid, and the pMD2.G envelope plasmid were transfected into 293T cells using the standard calcium phosphate transfection method. After 48 h, the lentivirus supernatant was collected for immediate use or frozen at −80 °C for later use. OSRC-2 and SW839 cells were infected with the lentiviral vectors to generate overexpressed TR4 and TR4-knockdown stable cell populations.

### Quantitative real-time PCR (qRT-PCR)

RNAs were extracted using Trizol reagent (Invitrogen, Grand Island, NY) and 1 μg of total RNA was subjected to reverse transcription using Superscript III transcriptase (Invitrogen). The qRT-PCR was then conducted using SYBR green by Bio-Rad CFX96 system. The expression of GAPDH mRNA level was used to normalize the expression level of a gene of interest using the 2^−ΔΔCt^ method. The primers for GAPDH were: Forward: 5′-GGA GCG AGA TCC CTC CAA AAT-3′; and Reverse: 5′-GGC TGT TGT CAT ACT TCT CAT GG-3′. The primers for TR4 were: Forward: 5′-TCC CCA CGC ATC CAG ATA ATC-3′; and Reverse: 5′-GAT GTG AAA ACA CTC AAT GGG C-3′.

### Western blot

Cells were lysed in RIPA buffer. Twenty micrograms proteins were loaded and then separated on 10% SDS/PAGE gel. PVDF membranes (Millipore, Billerica, MA) were then used for western transfer. After being blocked in 5% milk, the membranes were incubated with appropriate dilutions of specific primary antibodies against GAPDH (Santa Cruz, #sc-166574, Paso Robles, CA), β-actin (Abcam, #ab8227, San Diego, CA), TR4 (Abcam, #ab109301), or AXL (Abcam, #ab32828). After being incubated with HRP conjugated secondary antibodies, the blots were visualized using the ECL system (Thermo Fisher Scientific, Rochester, NY).

### RNA–RNA interaction prediction

Based on the established methods described in a previous paper [58], we computed the interaction lncRNA partners of the 5′ region of AXL mRNA. An ultrafast RNA–RNA interaction prediction system RIblast (https://github.com/fukunagatsu/Riblast) [59] was utilized on a lncRNA database (including 28,031 lncRNAs). An energy ranking list was generated and we picked up the top 100 lncRNAs.

### RNA Immunoprecipitation (RIP)

Cells were lysed in RIPA buffer supplemented with RNase and protease inhibitor cocktail. RNase-free DNase (New England Biolabs, Rochester, NY) (400 U) was then added to the lysate and incubated on ice for 30 min. To test protein–RNA interaction, 5 µg of TR4 antibody was added into 500 µl of the supernatant and the mixture was incubated at −4 °C overnight (normal rabbit IgG as control). To test RNA–RNA interaction, 500 µl of the supernatant was incubated with a final concentration of 1 µM biotinylated AXL RNA bait (5′-TGA CAC CGA AGG GTG CCC GTG AGT C-3′). Protein A/G beads (for protein–RNA interaction) were pre-blocked by 15 mg/ml BSA in PBS. Pierce^TM^ Streptavidin Agarose (Thermo Fisher Scientific^TM^, #20347) beads (for RNA–RNA interaction) were pre-blocked by 10 µM RNA from torula yeast Type VI (MilliporeSigma, #R6625, St. Louis, MO) in PBS. The pre-blocked beads were then added to the antibody/bait-lysate mixture and incubated for another 2 h at −4 °C. The RNA/antibody or RNA/RNA complex was washed four times before RNA extraction, which is the same as described in qRT-PCR section.

### Luciferase reporter gene assays

The 1.5 kb human lncTASR (ENST00000600671.1) promoter was cloned into pGL3 basic vectors (Promega, Madison, WI). Site-directed mutagenesis of the TR4 binding site in the lncTASR 5′ promoter was achieved by mutating part of the site to EcoRI cutting site (-GAATTC-). The DNA (pGL3 with wild type or mutant lncTASR promoter) was transfected into OSRC-2 cells using Lipofectamine 3000 (Invitrogen) according to the manufacturer's instruction. PRL-TK was also transfected as an internal control to normalize the transfection efficiency. After 36 h transfection, luciferase activity was measured by Dual-Luciferase Assay (Promega) according to the manufacturer’s instruction.

### Mouse model of orthotopic tumor implantation and drug administration

Six weeks old nude mice were purchased from NCI and injected with 1 × 10^6^ pWPI-OSRC-2-Luc cells (mixed with Matrigel, 1:1) under the left subrenal capsule. After 7 days tumor development, mice were randomized into four groups and treated every other day by intraperitoneal injection, the groups were (1) DMSO control (four mice), (2) tretinoin 25 mg/kg (six mice), (3) sunitinib 20 mg/kg (four mice), or (4) sunitinib 20 mg/kg + tretinoin 25 mg/kg (six mice). Tumor development and metastasis were monitored by Fluorescent Imager (IVIS Spectrum, Caliper Life Sciences, Hopkinton, MA) once a week. After 28 days’ treatments, the mice were sacrificed, and tumors and any metastases were removed for studies. Tumor weights were measured and calculated by the weight of the left kidney with tumor minus the weight of the right normal kidney from the same mouse. Sample size estimation was based on our previous similar animal model results [21]. If the mouse died before being sacrificed at a predetermined date, it was excluded. All the animal experiments were performed in accordance with the guidelines of the University of Rochester Medical Center Animal Care and Use Committee for animal experiments. The investigators were not blinded to group allocations during the experiment.

### Immunohistochemical (IHC)

Tissues were fixed in 10% (v/v) formaldehyde in PBS, embedded in paraffin, cut into 5 μm sections and used for IHC staining with human TR4 antibody (Abcam, #ab109301).

### Statistics

Data are presented as mean ± s.e.m. from at least three independent experiments. Two-sided tests were conducted by SPSS 17.0 (SPSS Inc., Chicago, IL). *p* < 0.05 was considered statistically significant.

## Supplementary information

Supplementary data.
