# Supplementary material for: Targeting the TR4 nuclear receptor-mediated lncTASR/AXL signaling with tretinoin increases the sunitinib sensitivity to better suppress the RCC progression
Source: Oncogene. 2019 Sep 9;39(3):530–45. doi: 10.1038/s41388-019-0962-8 (PMC6962095; doi:10.1038/s41388-019-0962-8)
Supplement: Supplementary file 1 — Supplementary data. [file 41388_2019_962_MOESM1_ESM.pdf]

Supplementary Data

Supplementary Fig. 1

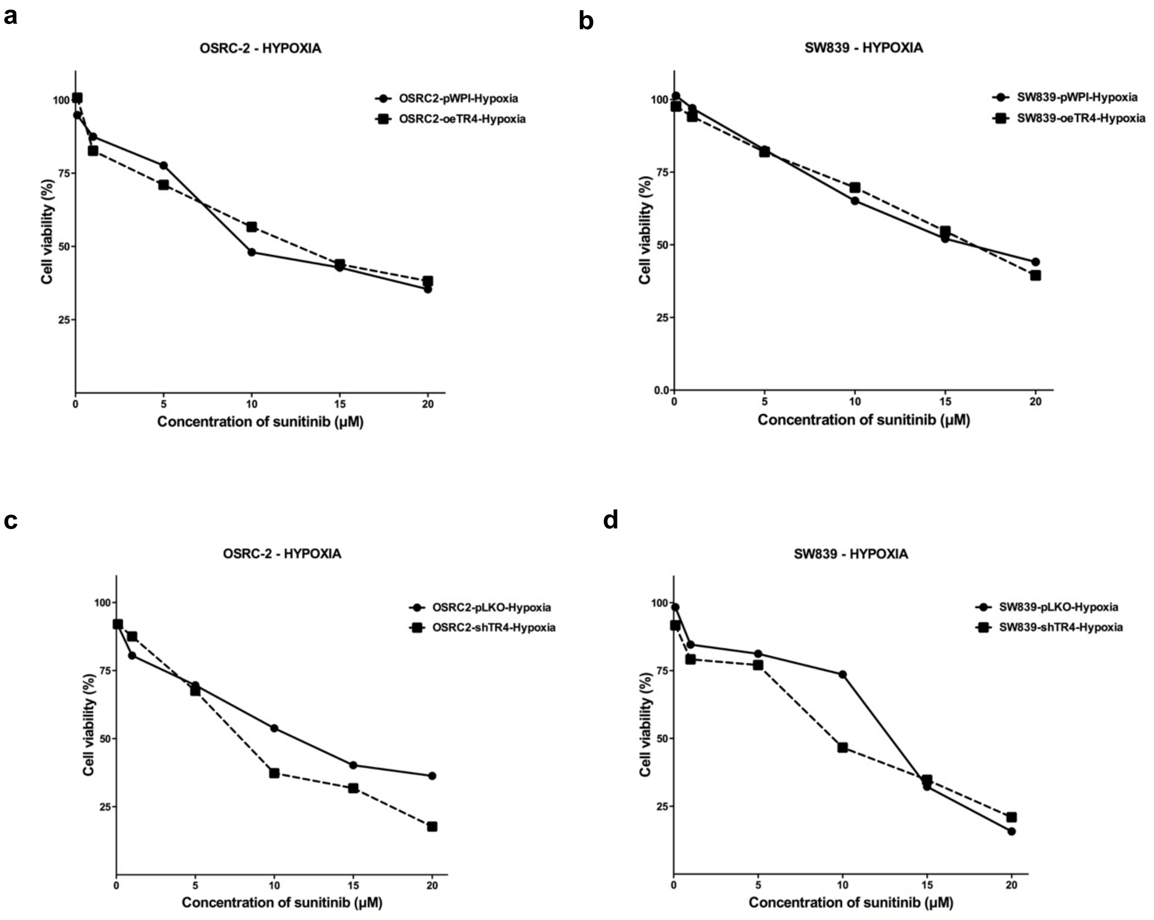

Supplementary Fig. 1 The influence of the combination of hypoxia treatment and regulating TR4 on the RCC sensitivity to Sunitinib.

(a) and **(b)**, After hypoxia treatment, the resistance to Sunitinib cannot be increased by introducing overexpressed TR4 in OSRC-2 **(a)** and SW839 **(b)** cells. The cells were cultured with different concentration gradients of Sunitinib and harvested after 24h treatment. **(c)** and **(d)**, After hypoxia treatment, the sensitivity to Sunitinib can be increased by knocking down TR4 in OSRC-2 **(c)** and SW839 **(d)** cells.

Supplementary Fig. 2

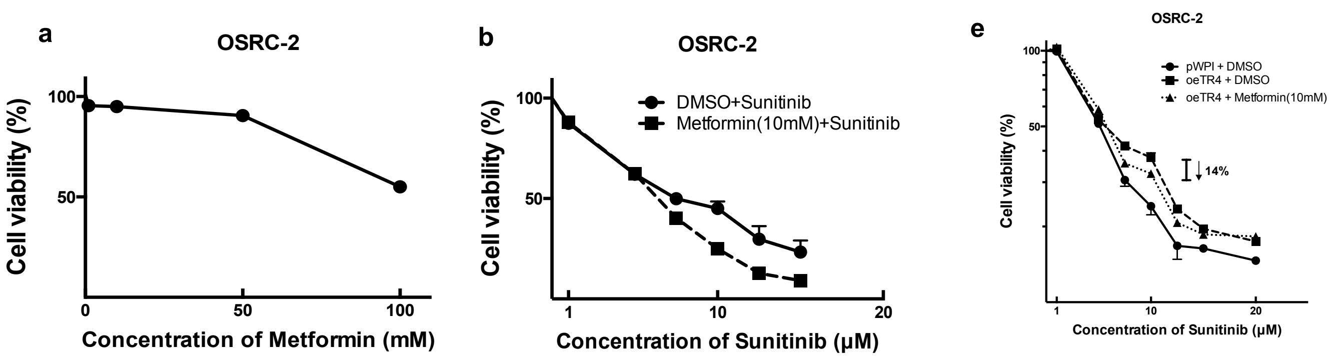

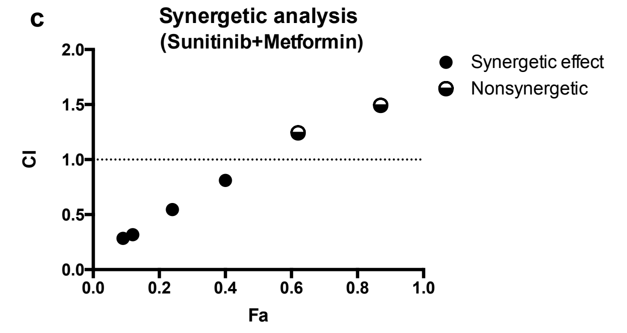

Sunitinib Dose (μM)	Metformin Dose (mM)	Fa	CI	Effect
1.0	10.0	0.87	1.49228	non
5.0	10.0	0.62	1.24167	non
7.5	10.0	0.40	0.81056	syn
10.0	10.0	0.24	0.54583	syn
12.5	10.0	0.12	0.31754	syn
15.0	10.0	0.09	0.28446	syn

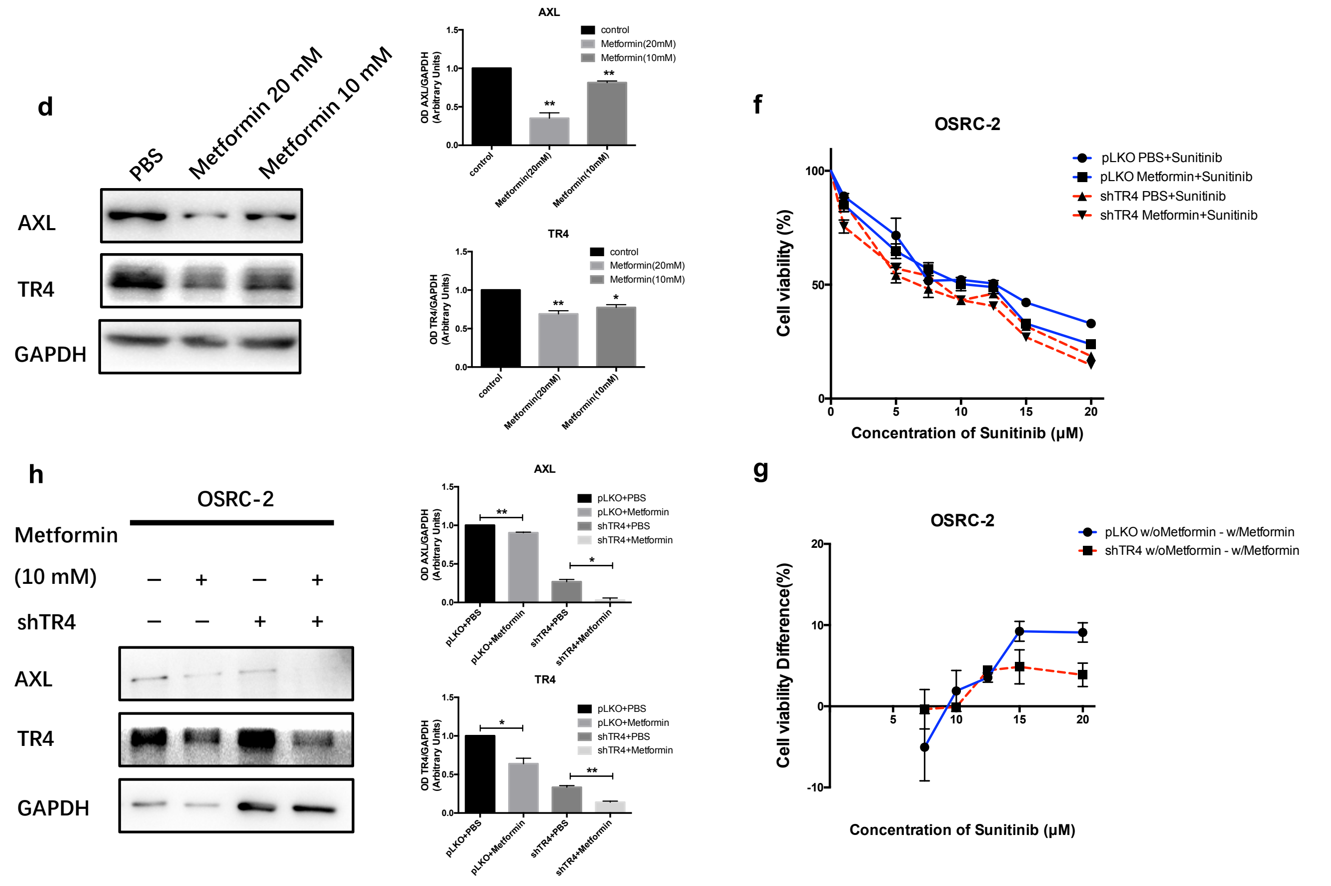

Supplementary Fig. 2 Metformin can increase Sunitinib sensitivity.

(a) Metformin alone was not toxic to RCC cells. **(b)** Metformin can enhance the sensitivity of RCC to Sunitinib when combined with high dose Sunitinib. **(c)** Metformin actually has synergetic effects when combined with Sunitinib in the treatment of RCC cells. **(d)** Western blotting (left) with quantification (right) shows that the expression of both TR4 and AXL decreased after treated with Metformin. **(e)** Metformin can reverse the resistance of RCC cells to Sunitinib caused by overexpressed TR4. **(f)** Viability of OSRC-2 cells in Sunitinib with/without Metformin and with/without TR4-shRNA. **(g)** DID (difference in difference) analysis shows that TR4 knockdown OSRC-2 cells have less difference change in Sunitinib-resistance with/without Metformin compared with OSRC-2 cells without TR4 knockdown. **(h)** Metformin decreases both TR4 and AXL protein expressions in OSRC-2 cells with pLKO vehicle and in TR4 knockdown OSRC-2 cells.

Supplementary Fig. 3

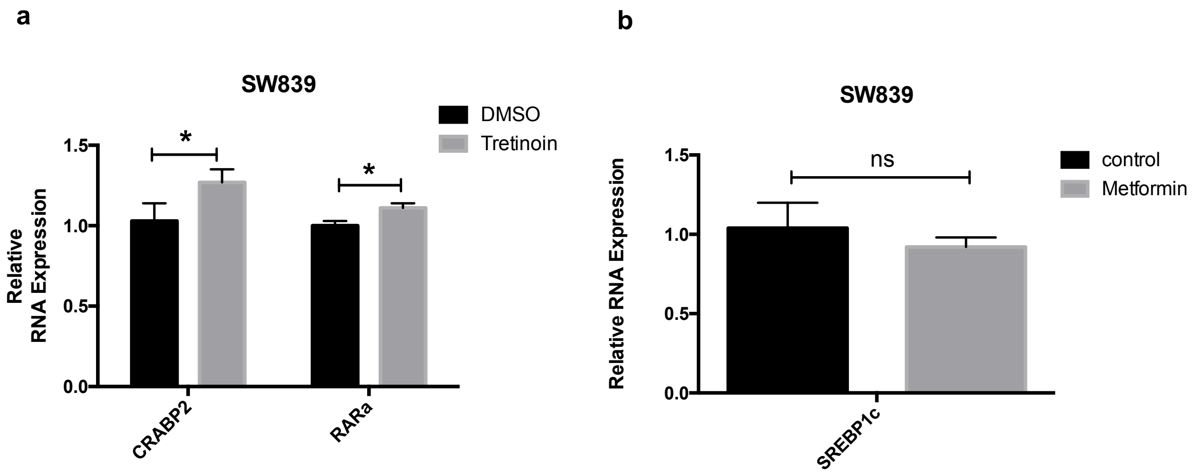

Supplementary Fig. 3 Verification of effects of Tretinoin and Metformin.

(a) Verification of effects of Tretinoin. Treated with Tretinoin, RNA expression of CRABP2 and RARα increase in SW839 cells. **(b)** Verification of effects of Metformin. Treated with Metformin, RNA expression of SREBP1c does not decrease significantly in SW839 cells.

Supplementary Fig. 4

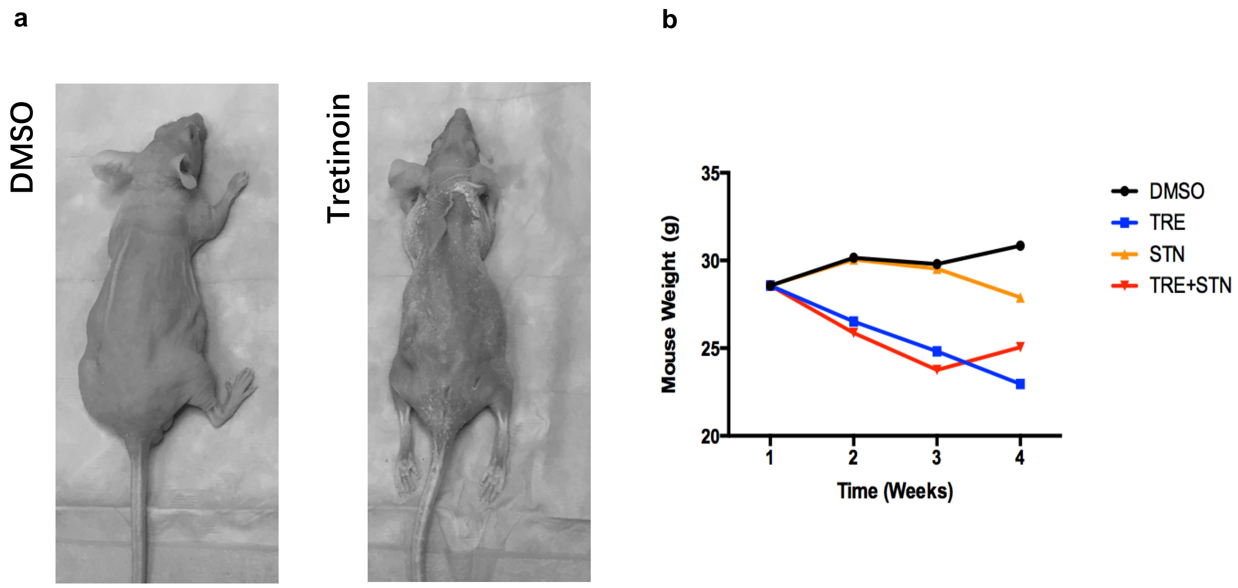

Supplementary Fig. 4 Tretinoin can cause weakness in nude mice.

(a), Representative photos showed that a mouse from the Tretinoin group looks weaker and less healthy than that from the DMSO group. **(b)**, The normalized mice weight curve shows that mice from the Tretinoin group or Sunitinib + Tretinoin group continuously lost weight throughout the treatment time course.
